# Manganese Oxide-Doped Hierarchical Porous Carbon Derived from Tea Leaf Waste for High-Performance Supercapacitors

**DOI:** 10.3390/ijms252010884

**Published:** 2024-10-10

**Authors:** Hsiu-Ying Chung, Hong-Min Chang, Chun-Pang Wang

**Affiliations:** Institute of Precision Electronic Components, College of Semiconductor and Advanced Technology Research, National Sun Yat-sen University, Kaohsiung 804201, Taiwan

**Keywords:** hierarchical porous materials, tea leaf biomass, manganese monoxide, supercapacitor, energy storage, sustainability

## Abstract

Hierarchical porous carbon derived from discarded biomass for energy storage materials has attracted increasing research attention due to its cost-effectiveness, ease of fabrication, environmental protection, and sustainability. Brewed tea leaves are rich in heteroatoms that are beneficial to capacitive energy storage behavior. Therefore, we synthesized high electrochemical performance carbon-based composites from Tie guan yin tea leaf waste using a facile procedure comprising hydrothermal, chemical activation, and calcination processes. In particular, potassium permanganate (KMnO_4_) was incorporated into the potassium hydroxide (KOH) activation agent; therefore, during the activation process, KOH continued to erode the biomass precursor, producing abundant pores, and KMnO_4_ synchronously underwent a redox reaction to form MnO nanoparticles and anchor on the porous carbon through chemical bonding. MnO nanoparticles provided additional pseudocapacitive charge storage capabilities through redox reactions. The results show that the amount of MnO produced is proportional to the amount of KMnO_4_ incorporated. However, the specific surface area of the composite material decreases with the incorporated amount of KMnO_4_ due to the accumulation and aggregation of MnO nanoparticles, thereby even blocking some micropores. Optimization of MnO nanocrystal loading can promote the crystallinity and graphitization degree of carbonaceous materials. The specimen prepared with a weight ratio of KMnO_4_ to hydrochar of 0.02 exhibited a high capacitance of 337 F/g, an increase of 70%, owing to the synergistic effect between the Tie guan yin tea leaf-derived activated carbon and MnO nanoparticles. With this facile preparation method and the resulting high electrochemical performance, the development of manganese oxide/carbon composites derived from tea leaf biomass is expected to become a promising candidate as an energy storage material for supercapacitors.

## 1. Introduction

Due to the growth and demand for consumer electronics and electric vehicles, the development of sustainable and cost-effective energy storage devices has become a top priority. A supercapacitor is a type of energy storage device with characteristics such as high power density, fast charge and discharge rates, and long-term cycle stability. They are particularly in demand for applications requiring bursts of energy over a short period of time. Based on their charge storage mechanism, supercapacitors are classified into two categories: electrical double-layer capacitors (EDLCs) and pseudocapacitors. The electrolyte ions rapidly adsorb and desorb onto the active surface at the electrode–electrolyte interfaces in EDLCs, whereas the charge is stored through the reversible faradaic redox reaction on the electrode surface in pseudocapacitors. Therefore, pseudocapacitors usually possess higher capacitance than EDLCs.

The most extensively studied electrodes for EDLCs are carbonaceous materials, including activated carbons [1,2], carbon nanotubes [3,4], carbon nanosheets [5,6], and graphene [7,8]. Among them, activated carbons are the most promising electrode materials for commercial applications due to their low fabrication costs, good electrical conductivity, excellent chemical stability, and high specific surface area.

Activated carbon produced from natural biomass is highly attractive from an economic and environmental perspective, not only because it can increase the value of the biomass or reduce the amount of biomass waste produced, but also because the biomass contains heterogeneous atoms that favor electrochemical properties. In particular, the multi-heteroatoms such as O, N, P, etc., intrinsically included in biomass can enhance the hydrophilicity and number of accessible electro-active sites of architecture-activated carbon [9,10,11,12]. Additionally, the inherently interconnected pore networks in biomass materials favor chemical activators to create more porosity, which also benefits electrochemical properties. To date, considerable attention has been focused on converting bio-waste, such as peanut shells [13,14], coconut shells [15], banana peels [16,17], and coffee grounds [18,19,20], into valuable activated carbon. Generally, biomass precursors undergo physical or chemical activation processes to create porosity, which is more beneficial to increasing overall electrochemical performance. Chemical activation is more commonly used than physical activation since it produces a relatively stable structure with good porosity and high specific surface area, in addition to lowering the activation temperature. However, the capacitance of activated carbons derived from biomass is typically in the range of 100–200 F/g at a current density of 1 A/g, which limits its application in high-power devices. Although increasing the specific surface area can increase the number of electrochemically active sites and thereby enhance the capacitive storage capacity, the structural stability of activated carbon dramatically decreases due to the porous microstructure. Another strategy to improve the electrochemical performance of carbonaceous materials is to introduce transition metal oxides, such as ruthenium oxide, iron oxide, cobalt oxide, nickel oxide, and manganese oxide, to form composites resulting from the additional pseudocapacitive behaviors, allowing fast faradaic redox reactions with the electrolyte [21,22]. Among various pseudocapacitive transition metal oxides, MnO_X_, in addition to its high capacitance, is abundant in nature, low cost, and environmentally friendly [23]. However, the non-optimized mass loading of the MnO_x_/activated carbon composites leads to severe degradation in electrochemical performance [23,24].

Tea is one of the most popular beverages in the world. Several billion tons of tea are consumed each year, and the majority of tea leaves are discarded after brewing. Tea residue contains polyphenols, lignin, hemicellulose, cellulose, amino acids, proteins, and vitamins. These substances are rich in nitrogen, oxygen, and phosphorus atoms, which are beneficial to capacitive energy storage. Although the fabrication process of one-step pyrolysis is simple, the produced biomass-derived carbon has limitations, such as a low specific surface area and numerous impurities [25]. Chemical activation is a simple and effective way to dramatically increase the specific surface area of biomass materials. Inal et al. and Thirumal et al. compared the effects of various chemical activators, including KOH, H_3_PO_4_, and K_2_CO_3_, on the microstructure and electrochemical performance of tea leaf residues [26,27]. In addition, the impregnation ratio of tea leaf residues and chemical activators is also a key experimental factor in creating hierarchical activated carbon with an applicable surface area and pore size distribution [25,28,29,30,31,32].

Designing electrode materials with rational compositions and superior microstructures is one of the key factors to achieve high-performance supercapacitors. Composite electrodes used in supercapacitors are usually fabricated through a physical mixture of biomass-derived activated carbon and transition metal oxides. Therefore, the entire synthesis process involves multiple steps in which activated carbon and transition metal oxides are synthesized separately and then mixed [24,33,34,35]. In contrast, in this study, we adopted a feasible one-pot approach in which activated carbon and transition metal oxides are produced synchronously. Peng et al. selected five common tea varieties in China and used their waste residues to synthesize electrodes for supercapacitors. Among them, the activated carbon derived from Tie guan yin tea exhibited excellent electrochemical behavior [36]. Therefore, brewed Tie guan yin tea leaves were used as raw materials in this study because the heteroatoms derived from organic compounds in tea leaves can serve as effective functional sites for carbonaceous materials. Firstly, Tie guan yin tea leaf waste was processed into hydrochar to achieve effective electrode materialization through the hydrothermal method. To increase the porosity and expand the specific surface area, the hydrochar was further chemically activated with KOH. In particular, various amounts of KMnO_4_ were incorporated into the KOH activation solution, which led to the synchronous loading of MnO nanoparticles onto hierarchical porous carbons. The results demonstrate that due to the synergistic effect between the electrochemical double-layer capacitance of biomass-activated carbon and the redox reaction pseudocapacitance of MnO, the highest specific capacitance of 337 F/g was achieved when the weight ratio of hydrochar:KOH:KMnO_4_ was 1:3:0.02.

## 2. Results and Discussion

**Figure 1:**
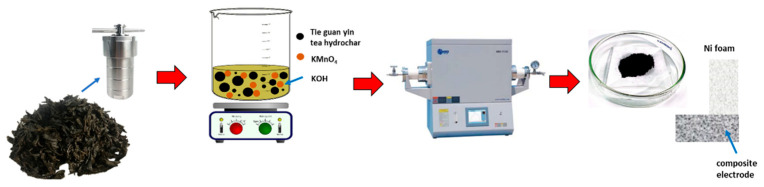
Schematic of the experimental process.

Figure 1 shows the schematic of the synthesis process for TGC specimens. Firstly, the Tie guan yin tea leaf powder undergoes a hydrothermal reaction. Subsequently, KOH and various amounts of KMnO_4_ are used to activate the dried hydrochar. Lastly, the mixture is calcined, neutralized, dried, and then prepared into electrodes.

**Figure 2:**
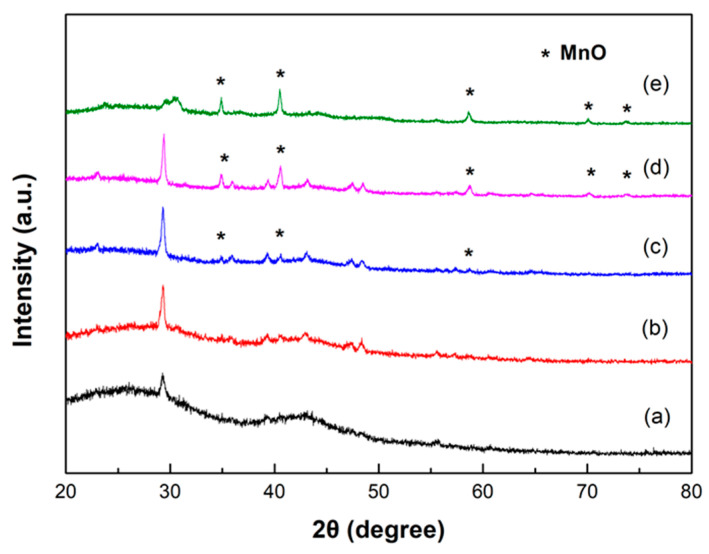
XRD patterns of (**a**) TGC0, (**b**) TGC1, (**c**) TGC2, (**d**) TGC3, and (**e**) TGC4.

Owing to the breaking and recondensation reactions during the calcination process, the carbon-rich components and protein in Tie guan yin tea leaf waste rearrange to finally produce amorphous carbon, as shown in Figure 2a. The two broad peaks centered at approximately 26° and 43° correspond to the (002) and (100) crystalline planes of disordered carbon. In addition, an obvious diffraction peak at 29.5° was also observed in other studies [27,30,32], which may be caused by the reaction of the trace substances contained in tea leaves with KOH or/and carbon during the impregnation process and high-temperature calcination. The above findings indicate that the TCG0 specimen exhibits turbostratic features and a low degree of graphitization. When KMnO_4_ was incorporated into the activation agent, a series of sharp diffraction peaks appeared at 34.7°, 40.3°, 58.3°, and 69.7°. These diffraction peaks are consistent with the (111), (200), (220), and (311) diffraction of cubic MnO of JCPDS 01-072-1533. As seen in Figure 2b–e, the diffraction intensity of the MnO peaks increased with the doping amount of KMnO_4_, revealing that the loading amount of MnO into the TGCx composites is proportional to the amount of KMnO_4_ added into the activation solution. In addition, the two broad diffraction peaks originating from the disordered carbon were suppressed from incorporating KMnO_4_. Abundant reducing volatiles, such as hydrogen and carbon monoxide, were released during the pyrolysis process, prompting the decomposition of KMnO_4_, thereby forming low-valence MnO instead of the most commonly obtained MnO_2_ [37]. The possible reaction mechanism is as follows [37]:

2KOH → 2K_2_O + H_2_O

K_2_O + C → 2K + CO

2K_2_O + CO_2_ → 2K_2_CO_3_

C + H_2_O → H_2_ + C

2K_2_O + H_2_ → 2K + H_2_

CO + H_2_O → H_2_ + CO_2_

2KMnO_4_ → K_2_MnO_4_ + MnO_2_ + O_2_

O_2_ + 2C → 2CO

MnO_2_ + C → MnO + CO

MnO_2_ + CO → MnO + CO_2_

MnO_2_ + H_2_ → MnO + H_2_O

Among the different manganese oxides (such as MnO, MnO_2_, Mn_2_O_3_, and Mn_3_O_4_), MnO is one of the most promising active materials for electrochemical capacitors because it has a high theoretical specific capacitance of approximately 1350 F/g, which is higher than the most widely studied material, MnO_2_ [37,38]. Nevertheless, the inferior cycling performance and rate capability of MnO, resulting from its lower electrical conductivity, limit its development [39,40].

**Figure 3:**
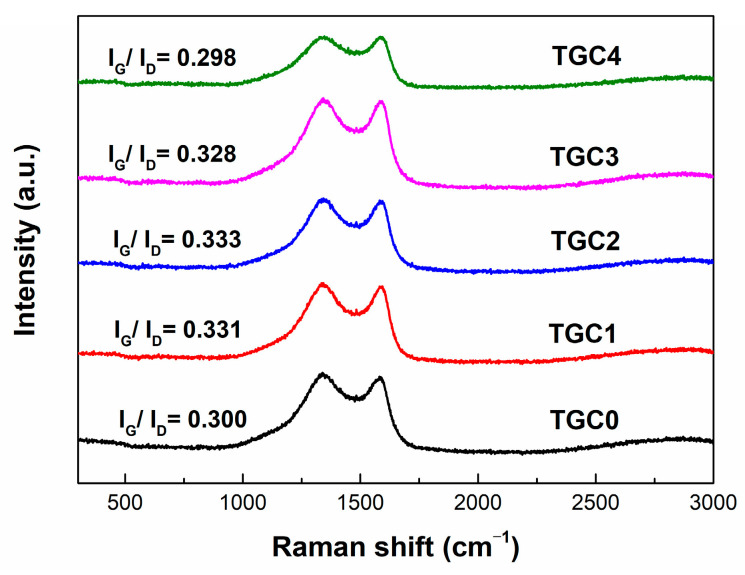
Raman spectra of TGC specimens.

The Raman spectra of TGCs shown in Figure 3 demonstrate two broad bands at around 1580 cm^−1^ (G-band) and 1320 cm^−1^ (D-band). The G-band corresponds to the C-C bond vibrations of carbon atoms with sp^2^ electronic configuration in the graphene sheet structure; in comparison, the D-band represents the disordered and imperfect structures in carbon materials. Typically, the intensity ratio of the G-band and D-band (I_G_/I_D_) is adopted to determine the graphitic degree of the carbonaceous materials [41,42]. Through the deconvolution of Raman spectra, the I_G_/I_D_ values were found to be 0.300, 0.331, 0.333, 0.328, and 0.298 for TGC0, TGC1, TGC2, TGC3, and TGC4, respectively. As only the KOH activator was used, TGC0 had a low I_G_/I_D_ value, indicating a high degree of disorder due to severe erosion damage of the strong base. The results also suggest that adding KMnO_4_ into the activation solution can enhance the graphitization degree of carbonaceous materials. More importantly, the TGC2 specimen exhibited the highest I_G_/I_D_ value of 0.333; in comparison, the values of TCG3 and TCG4 decreased, meaning that an appropriate incorporation amount of KMnO_4_ is required to achieve the highest degree of graphitization of the porous Tie guan yin tea leaf activated carbon. In contrast, the I_G_/I_D_ value of TGC4 was slightly lower than that of the control group, TGC0, which means that the formation and distribution of large amounts of MnO nanoparticles may disturb the internal ordering of Tie guan yin tea leaf activated carbon in the synthesis process.

**Figure 4:**
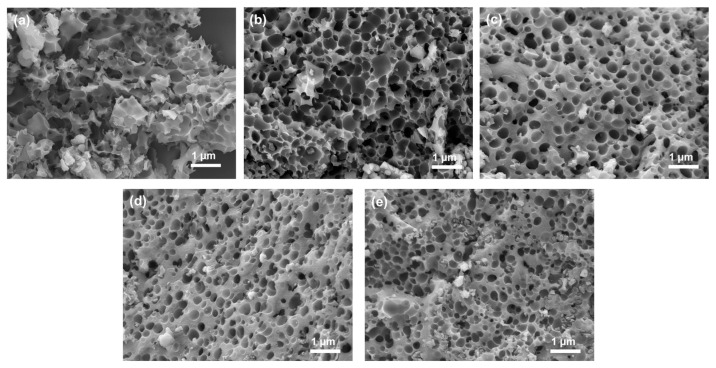
SEM images of (**a**) TGC0, (**b**) TGC1, (**c**) TGC2, (**d**) TGC3, and (**e**) TGC4.

The morphology of the as-prepared samples was investigated via SEM. As shown in Figure 4a, the KOH activator deeply penetrated the Tie guan yin tea leaf hydrochar and facilitated the formation of a highly etched morphology with abundant fragments and pore space, providing greater ion accessibility and space charge distribution. The developed microstructure was three-dimensional and randomly interconnected in appearance, with a variety of pore sizes ranging from a few nanometers to hundreds of nanometers in scale. As shown in Figure 4b, when KMnO_4_ was added to the KOH activator, the activated carbon was not eroded to the same degree as TGC0, indicating that KMnO_4_ slows down the erosion ability of KOH. Following the continuous increase in the concentration of KMnO_4_, the effect of inhibiting KOH erosion ability was more significant. The pore diameter appeared smaller, the three-dimensional hierarchical microstructure became less apparent, and the distance between the pores was longer, thus showing more uneroded flat areas. Notably, a large quantity of MnO nanoparticles with an approximate size of 50–100 nm attached to the erosive holes of the TGC2 specimen (Figure 4c). However, some accumulated MnO nanoparticles around the erosive hole were observed in the TGC3 specimen (Figure 4d). As the concentration of the incorporated KMnO_4_ was further increased, the phenomenon of MnO accumulation and agglomeration became obvious (Figure 4e). Some agglomerates even completely blocked the pore entrance, which may seriously impede electrolyte ions from entering the porous structure inside the carbon material to carry out charge reactions via the electric double-layer mechanism.

**Figure 5:**
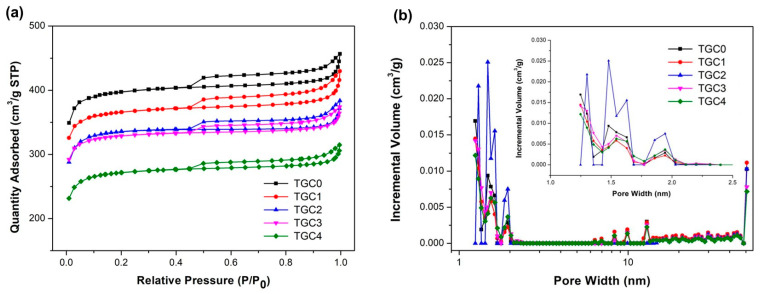
(**a**) N_2_ adsorption–desorption isotherms and (**b**) pore size distribution of TGC specimens.

The internal structure property and pore size distribution have significant effects on electrolyte ion transport. Nitrogen adsorption/desorption testing was utilized to investigate the surface area and associated pore structure of the as-prepared sample, as shown in Figure 5a. According to the IUPAC classification, the adsorption/desorption isotherms of all TGCs were classified as type IV models, where a steep N_2_ absorption at P/P_0_ < 0.1 and a typical H4 hysteresis loop at P/P_0_ > 0.45 were exhibited. This finding suggests that mesopores and micropores coexist and are network-connected to each other, thus forming a hierarchically connected pore-channel structure. The measured BET-specific surface areas of TGC0, TGC1, TGC2, TGC3, and TGC4 were 1483, 1367, 1259, 1229, and 1017 m^2^/g, respectively, indicating that higher KMnO_4_ doses facilitate the decrease in total specific surface area in addition to the decrease in micropore surface area. This finding may be attributed to the fact that, as observed in the SEM images, as more MnO crystals form, these nanoparticles accumulate and agglomerate, and some even block the pores of carbonaceous materials. Figure 5b demonstrates the pore size distribution calculated using the nonlocal density functional theory (NLDFT) model. Although the micropore volumes of TGCs were reduced from 0.5945 to 0.4043 cm^3^/g, compared with the other specimens, TGC2 had an obvious pore volume distribution in the pore diameter range of less than 2 nm. It is worth mentioning that TGC2 had the highest ratio of micropore volume to total volume (V_micro_/V_total_), in addition to the highest ratio of micropore surface area to total surface area (Table 1). The electrolyte ions can be effectively absorbed and transported in the electrode material with reasonable pore size distribution, where mesopores act as ion transport channels and micropores are preferred for ion adsorption and desorption [43,44].

**Figure 6:**
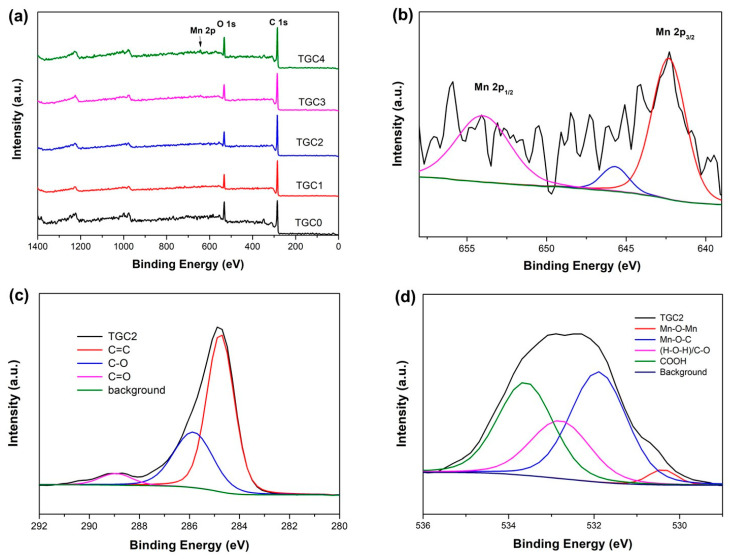
(**a**) Wide-scan XPS spectra of TGC specimens. High-resolution XPS spectra of TGC2 for (**b**) Mn 2p, (**c**) C 1s, and (**d**) O 1s.

Compared with TCG0, the XPS spectra of the other TGCx specimens with the loading of KMnO_4_ into activation solution exhibited an Mn 2p signal, excluding the C 1s and O 1s signals, as shown in Figure 6a. Two characteristic peaks at 642.3 and 653.9 eV with a spin energy separation of 11.6 eV corresponding to the spin-orbit doublets of Mn 2p_3/2_ and Mn 2p_1/2_, respectively, can be observed in the Mn high-resolution spectrum (Figure 6b and Figure S1) [37,45]. In particular, a satellite peak located near 645.6 eV was detected, which was ascribed to divalent manganese (Mn^2+^), confirming the formation of MnO [37,46] instead of the MnO_2_ reported in most studies. Moreover, this XPS Mn 2p analysis is consistent with the aforementioned XRD results.

The high-resolution C 1s spectrum (Figure 6c and Figure S2) can be deconvoluted into three individual peaks at 284.8, 286.1, and 288.9 eV, which are associated with the C=C, C-O, and C=O bonds [23,31,36], respectively, where C-O and C=O functional groups are incredibly beneficial to enhance the access of electrolyte ions. The proportions of deconvoluted carbon functional bonds are summarized in Table 2. The results demonstrate that TGC2 contains 37.15% C-O and C=O functional groups, the highest content ratio among all TGC specimens.

Moreover, the high-resolution O 1s spectrum (Figure 6d and Figure S3) of the TCG specimen with the incorporation of KMnO_4_ can be deconvoluted into four peaks at 530.4, 531.9, 532.7, and 533.7 eV, corresponding to the Mn-O-Mn, Mn-O-C, (H-O-H)/C-O, and COOH bonds, respectively. The appearance of a unique Mn-O-C bonding at 531.9 eV in the TCG1, TCG2, TCG3, and TCG4 samples indicates that the bridging between MnO and the activated carbon matrix takes place through chemical hybridization bonding rather than simple physical attachment only. Importantly, the formation of Mn-O-C bonds improves electrical contact, leading to higher rates of performance and better cycling stability [47]. Notably, as seen in Table 3, the content ratios of Mn-O-Mn and Mn-O-C bonds increase proportionally with the concentration of incorporated KMnO_4_, which implies that both the amount of MnO formed and the degree of chemical hybridization between MnO and Tie guan yin tea leaf activated carbon also increase. In addition, the content of functional groups (H-O-H)/C-O and COOH in the TGC2 specimen remains high, not reducing considerably due to the formation of MnO.

**Figure 7:**
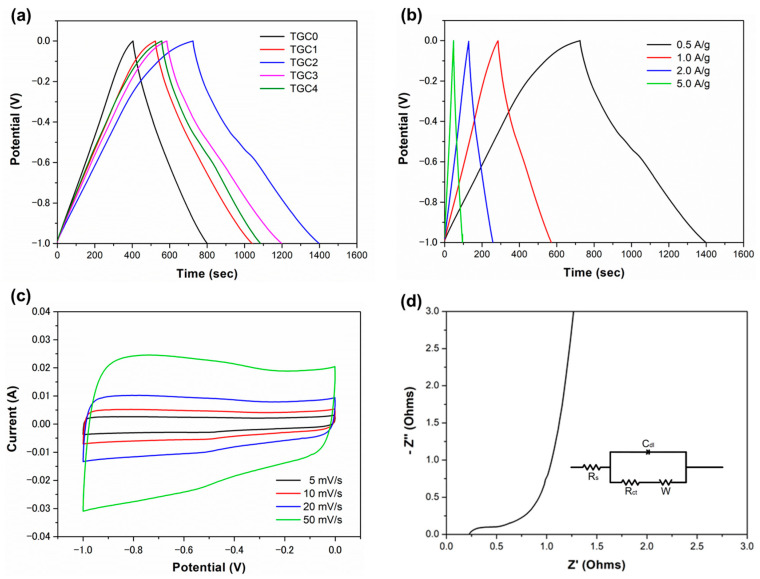
(**a**) GCD curves of TGC electrodes at a current density of 0.5 A/g. (**b**) GCD curves at current densities of 0.5, 1, 2, and 5 A/g, (**c**) CV curves at scan rates of 5, 10, 20, and 50 mV/s, and (**d**) EIS Nyquist plot of the TGC2 electrode.

Electrochemical assessments were carried out using a three-electrode configuration system through the analysis of cyclic voltammetry (CV), galvanostatic charge–discharge (GCD), and electrochemical impedance spectroscopy (EIS), with a potential window between −1 and 0 V in the 6 M KOH aqueous electrolyte. Figure 7a shows a comparison of the charge–discharge behavior of all TGC specimens at a constant current density of 0.5 A/g. The charge–discharge curve of TGC0 displays a quasi-isosceles triangle; in comparison, the slope of the discharge curve for the other TGC specimens is evidently lower than that of the TGC0 specimen, indicating a slower discharge rate and, therefore, a longer discharge time. The specific capacitances of the TGC0, TGC1, TGC2, TGC3, and TGC4 electrodes were calculated to be 198, 258, 337, 307, and 264 F/g, respectively. Among the specimens, TGC2 possessed the best charge–discharge electrochemical properties.

Furthermore, the charge–discharge behavior of TGC2 was examined at various current densities, as shown in Figure 7b. The specific capacitance values were 337, 286, 260, and 239 F/g at the current densities of 0.5, 1, 2, and 5 A/g, respectively. Except for the low current density of 0.5 A/g, the charge–discharge curves of TCG2 conducted under other high current densities are nearly symmetric and linear shapes, implying that a rapid and reversible reaction occurred on the electrode surface. In addition, no obvious IR drop appears in the discharge curve. The CV curves of the prepared TGC2 electrode at various scan rates ranging from 5 to 50 mV/s are presented in Figure 7c. The shapes of the CV curves are quasi-rectangular, and no distinct redox peaks are observed within the operating voltage range. This finding indicates that the primary electrical charge storage mechanism in the TGC2 composite electrode takes place through the electrical double layer in Tie guan yin tea leaf activated carbon; however, the redox reaction between MnO nanoparticles and the electrolyte dominates the overall charge storage capacity.

Moreover, the electrochemical behavior of the TGC2 electrode was further characterized via EIS, as depicted in Figure 7d. In the low-frequency region, the slope of the curve is high, indicating low resistance for electrolyte ion diffusion and approaching ideal charge storage capacitive behavior [32,36]. Nevertheless, the Nyquist plot in the high-frequency region exhibits a semicircle with a small diameter. The first intercept of the semicircle with the real axis (Z’) represents the solution resistance (R_s_), while the closed semicircle constitutes the interfacial resistance of charge transfer (R_ct_) [27,48]. The obtained R_s_ and R_ct_ of the TGC2 electrode were approximately 0.23 and 0.52 Ω, respectively, indicating high ionic conductivity and high charge transfer. The equivalent series resistance (ESR) is roughly equal to the diameter of the semicircle (R_ct_ − R_s_) [48]. The equivalent circuit diagram derived from the Nyquist plots is presented in the insert of Figure 7d. The TGC2 electrode demonstrates a low ESR value, implying higher electrical efficiency and less energy dissipation.

The three-dimensional and randomly interconnected microstructure of Tie guan yin tea leaf activated carbon produced by the KOH activator erosion provides higher electrolyte ion accessibility and more electric double-layer reaction sites, resulting in an improvement in capacitance. Meanwhile, MnO nanoparticles undergo redox reactions and contribute pseudocapacitive parts. However, when KMnO_4_ is incorporated into the KOH activator, although MnO nanoparticles are formed, the corrosive ability of KOH is also reduced. Therefore, the ratio of the synthesized MnO and Tie guan yin tea leaf activated carbon has pronounced effects on the electrochemical properties of the composite electrodes. If too much MnO is formed, the aggregation and accumulation of MnO nanoparticles will not only reduce the number of exposed active sites, but also deteriorate the conductivity and the graphitization degree of the composites, which is unfavorable in improving capacitive performance. In addition, the added amount of KMnO_4_ also affects the corrosive porosity, thus reducing the specific surface area and pore volume of the carbonaceous materials. Therefore, controlling the loading amount of KMnO_4_ in the KOH activator is a key factor. The results of previous studies show that manganese oxide clusters with relatively large sizes worsen the electrochemical properties of supercapacitors [49]. As shown herein, an appropriate amount of KMnO_4_ incorporation can keep the generated MnO in the state of nanoparticles, evenly distributing on the corrosive surface, in addition to maintaining a high micropore volume ratio (V_micro_/V_total_) and having a relatively high proportion of functional groups. Benefiting from the above characteristics, the TGC2 composite possesses the highest specific capacitance. Table 4 shows the comparison between tea waste-derived porous activated carbon materials and other porous carbon materials.

## 3. Materials and Methods

### 3.1. Materials and Chemicals

All chemicals used in this work were of analytical grade and were used as received without further treatment. The Tie guan yin tea leaves used in this study were purchased from TenRen’s TEA Company, Taiwan, China.

### 3.2. Preparation of Tie Guan Yin Tea Leaf Carbon (TGC)

The porous Tie guan yin tea leaf carbon (TGC) was prepared in the laboratory using a two-stage process. Firstly, the brewed waste Tie guan yin tea leaves were rinsed several times with clean water, dried at 80 °C for 12 h, and ground into fine powder. Thereafter, the fine Tie guan yin tea powder was mixed thoroughly with deionized water at a ratio of 1:15. The aqueous solution was placed in a stainless steel autoclave and maintained at 180 °C for 12 h for the hydrothermal reaction, followed by cooling to room temperature. The intermediate precipitates were sequentially filtered and then dried at 80 °C for 6 h. In the second stage, the resultant Tie guan yin tea hydrochar was chemically activated using KOH. To improve the energy storage capability of the carbon-based materials, KMnO_4_ was added to the KOH activation solution. The weight ratio of hydrochar:KOH:KMnO_4_ was 1:3:x, where x = 0, 0.01, 0.02, 0.03, and 0.04. After impregnation at 90 °C for 2 h, the aqueous activator was removed. The intermediate precursor was dried at 80 °C for 12 h, followed by transfer into a horizontal tube furnace at 750 °C for 2 h at a heating rate of 10 °C/min under N_2_ atmosphere with a flow rate of 100 mL/min. Thereafter, the calcined powder was ground in a mortar, neutralized with 1 M HCl aqueous solution, and washed with a sufficient amount of deionized water until its pH value reached 7 in order to remove the captured potassium compounds and other impurities. Finally, the cleaned powder was dried at 80 °C for 12 h. Based on the various ratios of the hydrochar, KOH, and KMnO_4_, the Tie guan yin tea leaf-based activated carbon samples were named TGC0, TGC1, TGC2, TGC3, and TGC4, where TGC0 was the specimen activated without KMnO_4_. The preparation weight ratios of TGC specimens are listed in Table 5.

### 3.3. Characterization

The obtained porous carbonaceous materials were degassed at 200 °C for 6 h, and their N_2_ adsorption–desorption isotherms were measured using a Micromeritics ASAP 2020 (Micromeritics, Norcross, GA, USA) at −196 °C. Specific surface area and pore size distribution were analyzed using Brunauer–Emmett–Teller (BET) and density functional theory (DFT) methods, respectively. Surface morphology and elemental composition were characterized by scanning electron microscopy (SEM, Zeiss (Oberkochen, Germany), Gemini 450) and dispersive X-ray spectrometry (EDS), respectively. The composition phase and bonding information were investigated via X-ray powder diffraction (XRD, Bruker (Karlsruhe, Germany), D8 Advance ECO) with a wavelength of 0.15406 nm from Cu K radiation and micro-Raman spectroscopy (Uni Nanotech Co., Ltd. (Yongin, Republic of Korea), UniDRON) with an Argon laser excitation wavelength of 532 nm, respectively. The surface chemical compositions and elemental states of the specimens were evaluated via X-ray photoelectron spectroscopy (XPS, Ulvac-Phi Inc. (Chigasaki, Japan), PHI VersaProbe).

### 3.4. Preparation of Electrode

The TGC powder, conductive carbon black, and polyvinylidene fluoride (PVDF) were thoroughly ground and mixed at a ratio of 8:1:1 in moderate N-methyl-2-pyrrolidone (NMP) solvent to form a homogeneous slurry and subsequently coated onto a pre-cleaned nickel foam mesh. The nanocomposite electrode was dried at 80 °C for 12 h, and the sample mass loading was configured to be approximately 1.5 mg/cm^2^.

### 3.5. Electrochemical Measurements

The electrochemical performances of the obtained samples were evaluated using a conventional three-electrode system comprising the TGC nanocomposite-coated nickel foam as a working electrode, a platinum sheet as a counter electrode, and a saturated calomel electrode (SCR) as a reference electrode in the 6 M KOH aqueous electrolyte. A Squidstat Plus Potentiostat/galvanostat (Admiral Instruments, Tempe, AZ, USA) electrochemical workstation was used to measure cyclic voltammetry (CV), constant current charge and discharge (GCD), and electrochemical impedance spectroscopy (EIS). The CV and GCD measurements were carried out in the potential range from −1 to 0 V at various scan rates ranging from 5 to 50 mV/s, and current densities ranging from 0.5 to 5 A/g, respectively. Moreover, the EIS measurements were carried out in the frequency range of 0.01 to 100 kHz with an amplitude of 10 mV. The capacitance C (F/g) of the single electrode was calculated from the GCD measurements according to the following equation [50,54].

(1) $$C=\frac{I\times ∆t}{m\times ∆V}$$

where I (A) is the constant discharge current, ∆t (s) is the discharge time, m (g) is the mass of active material on the single electrode, and ∆V (V) is the potential change.

## 4. Conclusions

Generally, the preparation of activated carbon and transition metal oxide composite electrodes is extremely time-consuming, since the individual materials need to be synthesized separately and then physically mixed. In contrast, in the present study, hierarchically porous Tie guan yin tea leaf-derived carbon/manganese monoxide composites were prepared through a facile one-step method, in which KMnO_4_ was incorporated into the KOH chemical activator, thereby allowing for the synchronous activation of Tie guan yin tea leaf hydrochar and the loading of manganese monoxide. The activator KOH effectively created the interconnected hierarchical porous structure inside the biomass materials, not only providing a large specific surface area to promote ion accessibility, but also shortening the ion diffusion distances. In addition, the simultaneously produced MnO nanoparticles from the redox reaction played a key role in providing pseudocapacitive characteristics. Although more KMnO_4_ was added, more MnO was generated, and strong Mn-O-C chemical bonding was formed between porous carbon and MnO. Our results show that when too much KMnO_4_ is added, the resulting MnO nanoparticles aggregate near the outer surface of the pore structure. In particular, the amount of KMnO_4_ added also affects the erosion capability of KOH, the crystallinity, and the graphitic degree of Tie guan yin tea leaf-derived carbonaceous materials. The sample prepared with a weight ratio of KMnO_4_ to hydrochar of 0.02 exhibited the highest I_G_/I_D_ value of 0.333, the highest ratio of micropore volume to total volume (V_micro_/V_total_), and high functional group content. Additionally, the TGC2 specimen demonstrated a high specific capacitance of 337 F/g at a current density of 0.5 A/g when evaluated with a three-electrode system in the 6 M KOH electrolyte, attributed to the synergistic effects between electrochemical double-layer capacitance from the Tie guan yin tea leaf-derived-porous carbons and faradic pseudocapacitive contribution from MnO.

## Figures and Tables

**Table 1:** Textural characteristics of TGC specimens.

Specimen	S_BET_ m^2^/g	S_micro_ m^2^/g	V_total_ cm^3^/g	V_micro_ cm^3^/g	Average Pore Diameter nm	S_micro_/S_total_ %	V_micro_/V_total_ %
TCG0	1483	1432	0.6882	0.5945	1.86	96.56	86.38
TCG1	1367	1320	0.6431	0.5477	1.88	96.56	85.17
TCG2	1259	1226	0.5747	0.5058	1.83	97.38	88.01
TCG3	1229	1193	0.5633	0.4941	1.84	97.07	87.72
TCG4	1017	976	0.4730	0.4043	1.87	95.97	85.48

**Table 2:** The XPS C 1s deconvoluted ratio of TGC specimens.

Specimen	C=C 284.8 eV	C-O 286.1 eV	C=O 288.9 eV	(C-O) + (C=O)
TCG1	75.04%	17.81%	7.15%	24.96%
TCG2	62.85%	31.72%	5.43%	37.15%
TCG3	63.68%	29.94%	6.38%	36.32%
TCG4	76.13%	18.97%	4.90%	23.87%

**Table 3:** The XPS O 1s deconvoluted ratio of TGC specimens.

Specimen	Mn-O-Mn 530.4 eV	Mn-O-C 531.9 eV	(H-O-H)/C-O 532.7 eV	COOH 533.7 eV
TCG1	2.04%	40.87%	31.62%	25.48%
TCG2	2.87%	40.71%	22.56%	33.86%
TCG3	4.10%	42.41%	27.15%	26.34%
TCG4	5.87%	53.06%	25.49%	15.58%

**Table 4:** Comparison of the tea waste-derived porous carbon materials and other porous carbon materials.

Active Materials	Current Density (A/g)	Specific Capacitance (F/g)	References
Microporous and mesoporous K_2_CO_3_-activated carbons produced from tea waste.	1.5 mA/cm^2^	203	[26]
KOH-activated biomass carbon from tea leaves.	0.5	132	[27]
KOH-activated porous carbons derived from tea waste.	0.05	256	[31]
Hierarchical porous carbon with multi-heteroatom co-doping from tea waste.	0.5	170	[32]
KOH-activated carbons derived from Bi luo chun tea leaf waste.	1	330	[36]
KOH-activated rod-like porous carbon from tea-waste.	1	332	[42]
Green tea waste-derived ultrathin mesoporous graphitic carbon nanoflakes.	0.5	165	[50]
Tea waste-derived microporous KOH-activated carbon.	1	170	[51]
Manganese oxide-doped hierarchical porous carbon derived from tea leaf waste.	0.5	337	This work
MnO_X_-modified corrugated carton-derived hierarchical porous carbon.	2.5	279	[23]
MnO_2_ and banana peel-derived 3D porous carbon composites.	0.3	140	[24]
Manganese oxide/soybean straw porous carbon composite	1	438	[35]
litchi shell-derived hierarchically porous carbon/MnO nanosheets.	1	496	[37]
MnO-decorated Salvinia adnata-based carbon.	1	437	[52]
Bamboo-based activated carbon @ MnO_2_ nanocomposites.	1	221	[53]

**Table 5:** The preparation weight ratios of TGC specimens.

Specimen	Tie Guan Yin Tea Hydrochar	KOH	KMnO_4_
TGC0	1	3	0
TCG1	1	3	0.01
TCG2	1	3	0.02
TCG3	1	3	0.03
TCG4	1	3	0.04

## Data Availability

Data is contained within the article and Supplementary Materials.

## Supplementary Materials

The following supporting information can be downloaded at https://www.mdpi.com/article/10.3390/ijms252010884/s1.
