# Supplementary material for: Manganese Oxide-Doped Hierarchical Porous Carbon Derived from Tea Leaf Waste for High-Performance Supercapacitors
Source: Int J Mol Sci. 2024 Oct 10;25(20):10884. doi: 10.3390/ijms252010884 (PMC11508140; doi:10.3390/ijms252010884)
Supplement: Supplementary file 1 [file ijms-25-10884-s001.zip › ijms-3215158-supplementary.pdf]

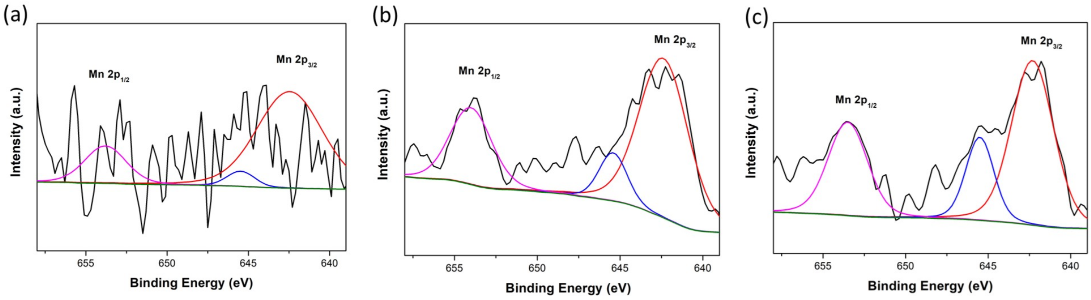

Figure S1. XPS high-resolution Mn 2p spectra of (a) TGC1, (b) TGC3, and TGC4.

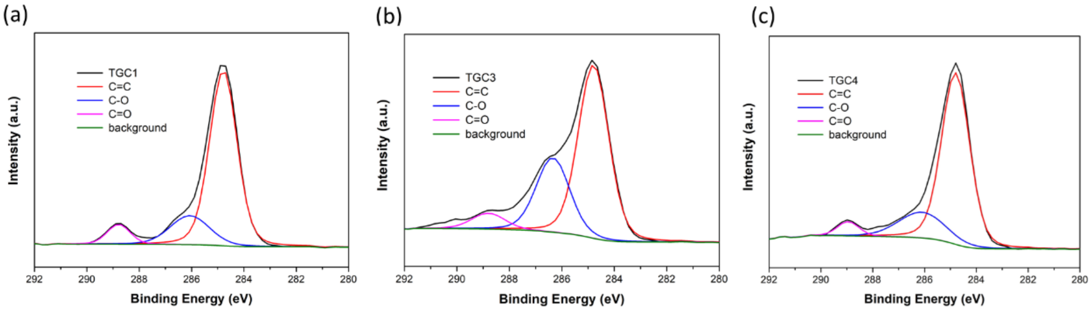

Figure S2. XPS high-resolution C 1s spectra of (a) TGC1, (b) TGC3, and TGC4.

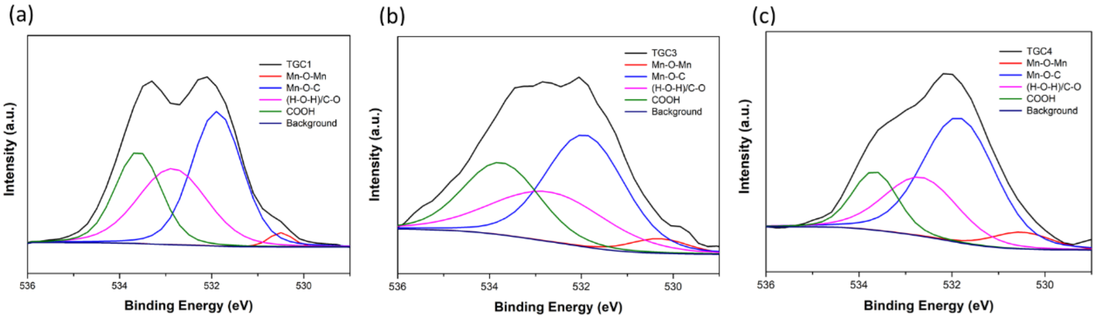

Figure S3. XPS high-resolution O 1s spectra of (a) TGC1, (b) TGC3, and TGC4.
